# A Facile Synthesis of Flower-like Iron Oxide Nanoparticles and Its Efficacy Measurements for Antibacterial, Cytotoxicity and Antioxidant Activity

**DOI:** 10.3390/pharmaceutics15061726

**Published:** 2023-06-14

**Authors:** Nazish Tabassum, Virendra Singh, Vivek K. Chaturvedi, Emanuel Vamanu, Mohan P. Singh

**Affiliations:** 1 Centre of Biotechnology, University of Allahabad, Prayagraj 211002, India; 2 Centre for Interdisciplinary Research in Basics Sciences, Jamia Millia Islamia, New Delhi 110025, India; 3 Department of Gastroenterology, Institute of Medical Sciences, Banaras Hindu University, Varanasi 221005, India; vkchaturvedi.nbt@gmail.com; 4 Faculty of Biotechnology, University of Agricultural Sciences and Veterinary Medicine of Bucharest, 011464 Bucharest, Romania

**Keywords:** Fe_2_O_3_ nanoparticles, co-precipitation, cancer cell line, cytotoxicity, antibacterial, antioxidant

## Abstract

The objective of this study was to investigate the rhombohedral-structured, flower-like iron oxide (Fe_2_O_3_) nanoparticles that were produced using a cost-effective and environmentally friendly coprecipitation process. The structural and morphological characteristics of the synthesized Fe_2_O_3_ nanoparticles were analyzed using XRD, UV-Vis, FTIR, SEM, EDX, TEM, and HR-TEM techniques. Furthermore, the cytotoxic effects of Fe_2_O_3_ nanoparticles on MCF-7 and HEK-293 cells were evaluated using in vitro cell viability assays, while the antibacterial activity of the nanoparticles against Gram-positive and Gram-negative bacteria (*Staphylococcus aureus*, *Escherichia coli*, and *Klebsiella pneumoniae*) was also tested. The results of our study demonstrated the potential cytotoxic activity of Fe_2_O_3_ nanoparticles toward MCF-7 and HEK-293 cell lines. The antioxidant potential of Fe_2_O_3_ nanoparticles was evidenced by the 1,1-diphenyl-2-picrylhydrazine (DPPH) and nitric oxide (NO) free radical scavenging assays. In addition, we suggested that Fe_2_O_3_ nanoparticles could be used in various antibacterial applications to prevent the spread of different bacterial strains. Based on these findings, we concluded that Fe_2_O_3_ nanoparticles have great potential for use in pharmaceutical and biological applications. The effective biocatalytic activity of Fe_2_O_3_ nanoparticles recommends its use as one of the best drug treatments for future views against cancer cells, and it is, therefore, recommended for both in vitro and in vivo in the biomedical field.

## 1. Introduction

Nanotechnology became a game-changing field in technology development in recent years. Among the various nanomaterials, nanoparticles received significant attention due to their unique physical and chemical properties, such as low melting point, specific magnetization, higher surface area, and specific optical properties [1]. These size-dependent properties and minimal harmful effects make them superior and suitable candidates in different areas of human activities [2]. Nanobiotechnology is currently gaining cumulative importance in the fields of nanomedicine, drug delivery, and immunology. Many new promising techniques and methods for synthesizing nanoparticles are being developed through chemical modification, biological reduction, and scaffolding to expand the application of nanobiotechnology in the biomedical field [3,4]. In recent years, various nanomaterials were synthesized by chemical and green synthesis methods. Some researchers used green chemical approaches for the synthesis of metallic nanoparticles. Ullah et al. reported the green synthesis of silver oxide (Ag_2_O) nanoparticles using leaves extract of *Parieteria alsinaefolia* as a reducing agent. Furthermore, various biological application was carried out to evaluate the efficiency of these nanoparticles [5]. In another study, researchers successfully synthesized a composite of silver-graphene nanoparticles ((Ag)_1_ − x(GNPs)x) via ex situ approach. The composite nanoparticles showed strong anticancer and antifungal properties [6]. Haris and their co-workers synthesized iron oxide nanoparticles from *Oscillatoria limnetica* extract and investigated the biomedical potential of these nanoparticles [7]. Among these nanomaterials, iron oxide nanoparticles (IONPs) catch an attraction by the scientific communities for their unique magnetic properties [8]. Due to their low toxicity and good biocompatibility, IONPs are considered to be the most favorable candidate for bioengineering and biomedical application such as bioimaging, targeted drug delivery, magnetic fluid hyperthermia (MFH), theranostics, detection of biological entities, biosensors, and photoablation therapy [9,10]. For various fundamental and biomedical uses, recent years saw a systematic study of various polymorphs of magnetic NPs such as Fe_3_O_4_, α-Fe_2_O_3_, γ-Fe_2_O_3_, and FeO [11,12,13]. Among them, hematite (α-Fe_2_O_3_) is more stable with optical, magnetic, and anticorrosive properties as well as outstanding chemical stability and biocompatibility which is beneficial in different technological applications [14]. These properties of hematite are used in innovative nanomaterial applications such as water splitting, water purification, gas sensing, solar energy conversion, catalyst, and anticorrosive agents [15]. Due to their attractive features, such as good stability, biocompatibility, low cost, and non-toxicity, these nano-sized Fe_2_O_3_ nanomaterials are more suitable for biomedical applications [16].

Many studies on the synthesis of Fe_2_O_3_ nanoparticles were conducted in recent years, detailing efficient synthesis methods for size control, stability, biocompatibility, and monodispersed iron oxide nanoparticles [17,18,19]. Among them, the coprecipitation process, one of the most popular techniques, was frequently used to synthesize Fe_2_O_3_ nanoparticles. Coprecipitation is a straightforward, affordable procedure that is carried out in comfortable settings without the use of any hazardous solvents [20]. The synthesis process is dependent on several variables, including pH, temperature, the type of salts used, and ionic strength, to produce nanoparticles that are the ideal size and form [21,22,23].

After knowing about the significance of Fe_2_O_3_ nanoparticles in biomedical areas, the current research was carried out to synthesize the nanoparticles using a practical co-precipitation approach. Then, using a variety of characterization, physicochemical screening of the produced nanoparticles was performed. Regarding this, human breast cancer (MCF-7) and human embryonic kidney (HEK-293) cell lines were used in the cytotoxic assessment of Fe_2_O_3_ nanoparticles. Additionally, the effectiveness of Fe_2_O_3_ nanoparticles against three different bacteria species—*Staphylococcus aureus* (*S. aureus*), *Klebsiella pneumoniae* (*K. pneumoniae*), and *Escherichia coli* (*E. coli*)—was evaluated. The antibacterial ability of human pathogenic bacteria, and cytotoxicity effects against cancer cell lines, suggest the potential of Fe_2_O_3_ nanoparticles for biomedical applications.

## 2. Materials and Methods

### 2.1. Chemicals and Reagents

We bought ammonia solution (25%) and ferrous sulphate heptahydrate (FeSO_4_.7H_2_O) from Sisco Research Laboratories (SRL) Pvt. Ltd. in India. The following items were acquired from Sigma-Aldrich, India: i.e., dimethyl sulfoxide (DMSO), antibiotics, Dulbecco’s Modified Eagle’s Medium (DMEM), foetal bovine serum (FBS), trypsin/EDTA, Dulbecco’s phosphate-buffered saline (DPBS), and 3-(4,5-dimethylthiazol-2-yl)-2,5-diphenyltetra Sodium nitroprusside. 1, 1-diphenyl, 2-picryl hydrazyl (DPPH) was purchased from Invitrogen in India. Double distilled water (DI) was used in the studies as a standard solvent and for washing.

### 2.2. Synthesis of Fe_2_O_3_ Nanoparticles

**Figure 1:**
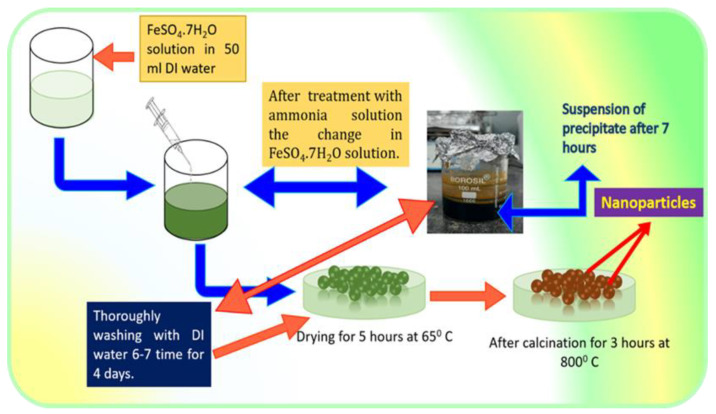
Fe_2_O_3_ nanoparticles are an emerging player in biomedical applications. Among various synthetic methods, the co-precipitation method appears to be the most successful method for batch production of Fe_2_O_3_ nanoparticles. The present diagrammatic illustration represents the facile and low-cost synthesis of Fe_2_O_3_ nanoparticles by modifying the coprecipitation route for better control of particle size.

Fe_2_O_3_ nanoparticles were synthesized from a simple low-cost coprecipitation route. Then, 0.1 M FeSO_4_.7H_2_O was dissolved in 50 mL DI water and stirred on a magnetic stirrer vigorously for 45 min. After that, the ammonia solution was added drop by drop under continuous stirring until the pH reached ~12 and the green color precipitate was formed. The solution was placed on magnetic stirring for 1–2 h. Afterward, the solution was removed from the magnetic stirrer until it settled down. The precipitate was washed thoroughly with DI water 2–3 times and dried at 65 °C for 5 h and calcinated at 800 °C for 3 h [20,24]. The synthesis process of Fe_2_O_3_ nanoparticles is represented in Figure 1.

### 2.3. Instrumentation

UV-visible spectrophotometer (SPECORD 210 PLUS double beam spectrophotometer, Analytic Jena, Germany) was used at room temperature, and the absorption band of the synthesized sample was measured with a resolution of 1 nm. The manufactured powder sample’s phase composition and degree of crystallinity were examined using an XRD diffractometer (Bruker AXSD8) and Cu-K radiation (=1.5406). The sample’s FTIR spectra were captured using a Perkin-Elmer 1600 Fourier transform instrument in the KBr pellet mode between 500 and 4000 cm^−1^. The morphology and chemistry of nanoparticles were studied using a field emission scanning electron microscopy (FESEM) FEI Quanta 200 F SEM, FEI Company Netherlands instrument with an EDX detector. Transmission electron microscopy with high resolution (HRTEM) (FEI Tecnai TF20) was used for TEM and selected area electron diffraction (SAED) analysis.

### 2.4. Evaluation of the Cytotoxic Activity of Fe_2_O_3_ Nanoparticles

#### 2.4.1. Cell Culture

Both the human embryonic kidney (HEK-293) and human breast cancer (MCF-7) cell lines were obtained from the NCCS in Pune, India, and were subcultured in a lab. The cell lines were grown in DMEM, a high glucose medium, which also contained 10% FBS and 2% penicillin/streptomycin. The culture was kept in an incubator with 5% CO_2_ at a temperature of 37 °C.

#### 2.4.2. MTT Assay

The 3-(4,5-dimethylthiazol-2-yl)-2,5-diphenyltetrazolium bromide (MTT) assay was performed with the Mosdam approach [25]. The culture media were supplemented with the diluted stock solution of Fe_2_O_3_ nanoparticles. Two distinct 96-well culture plates were seeded with 1 × 10^5^ MCF-7 and HEK-293 cell lines per well and incubated at 37 °C overnight. The cells were treated with various doses of Fe_2_O_3_ nanoparticles (25, 50, 75, 100, and 125 µg/mL) the next day and were incubated for 24 h at 37 °C with 5% CO_2_. The cells that were not exposed served as a control. MTT solution (10 µL) was added to each well after 24 h, and the culture plates were then incubated at 37 °C for 3–4 h. Formazan crystals were dissolved in 100 mL of DMSO following incubation and a reading at 570 nm of the absorbance was taken. The experiment was performed three times, the cell viability percentage was determined using the following formula, and the mean average value was obtained from triplicates.

$$cell\;viability\;(\%)=\frac{\left(Absorbance\;of\;treated\;cells\right)}{\left(Absorbance\;\;of\;control\;cells\right)}\times 100$$

### 2.5. Antibacterial Activity of Fe_2_O_3_ Nanoparticles

The antibacterial study of the Fe_2_O_3_ nanoparticles was executed against three different pathogenic bacteria *E. coli* (ATCC-25922), *K. pneumoniae* (ATCC-31488), and *S. aureus* (ATCC-25323) by the disc diffusion method using Kasithevar et al. procedure with some modification [26]. These bacteria were cultured for 24 h in nutrient broth. Afterward, 100 μL (106 CFU/mL) of the bacterial strains was placed on the agar plates to make the culture turf. The Fe_2_O_3_ nanoparticles were mixed in DMSO to make a stock solution of 1 mg/mL. Later on, 10, 20, 30, and 40 μg/mL of sample disc of nanoparticle were placed on a nutrient agar media plate along with the control (DMSO). The plates were placed in the incubator for 24 h and the respective inhibition zone (mm) for bacterial species were measured. The experiment was performed in triplicates.

### 2.6. Antioxidant Activity of Fe_2_O_3_ Nanoparticles

The free radical scavenging activity of the Fe_2_O_3_ nanoparticles was carried by using 1,1-diphenyl-2-picryl hydrazyl (DPPH) assay. Kurechi and their co-worker’s method was followed with minor modifications [27]. Briefly, a stock solution of 2 mg/mL of the Fe_2_O_3_ nanoparticles and standard solutions of L ascorbate was prepared and diluted to obtain desired concentrations. After that, an equal amount of the diluted solutions and DPPH (0.05 mg/mL) solution was mixed and incubated for 30 min. The experiments were conducted at room temperature. The absorbance was recorded at 517 nm using a spectrophotometer. The scavenging activity was calculated as

$$Scavenging\;(\%)\;=\;\frac{A_{c}-A_{s}}{A_{c}}\times 100;$$

where *A_c_*-absorbance of the control sample (DPPH) and *A_S_*-absorbance of a sample with DPPH. The experiment was executed in triplicates. Additionally, 0.1 mM DPPH and L-ascorbate were used as a control and standard solution.

The Griess reaction was used to measure the nitric oxide scavenging activity. After oxygen interacts with sodium nitroprusside in a solution at a physiological pH, nitric oxide is produced. Then, 5 mM sodium nitroprusside was mixed with 3 mL of various concentrations of Fe_2_O_3_ nanoparticles and standard solution L ascorbate in phosphate buffer (pH 7.4) to conduct the nitric oxide assay. Additionally, the solution mixture was left to stand for 30 to 40 min. Then, 1.5 mL of the incubated solution and Griess reagent were combined after the incubation period and left to stand for 30–35 min. A spectrophotometer was used to measure the mixture’s absorbance at 540 nm. Using the absorbance values of the mixture in comparison to the control solution, an estimate of the percentage of Fe_2_O_3_ nanoparticles’ nitric oxide scavenging activity was made.

### 2.7. Statistical Analysis

The one-way ANOVA statistical analysis was carried out with the help of the software Graph Pad Prism v5.0 (San Diego, CA, USA). The differences were deemed significant at *p* < 0.05. Mean ± standard deviation (mean ± S.D.) is used to represent all of the data.

## 3. Results and Discussion

There are two well-known crystalline of Fe2O_3_: maghemite (the γ-phase) with a cubic structure and hematite (the α-phase) with a rhombohedral structure. According to studies, the phase transformation occurs during calcination at a higher temperature (800 °C) that results in the transformation of α-Fe_2_O_3_ powder which underwent crystalline structure from an amorphous state [28]. Furthermore, we examined the synthesized Fe_2_O_3_ nanoparticles by different characterization techniques to obtain information on their physical and chemical properties.

### 3.1. Physicochemical Characterization of Fe_2_O_3_ Nanoparticles

**Figure 2:**
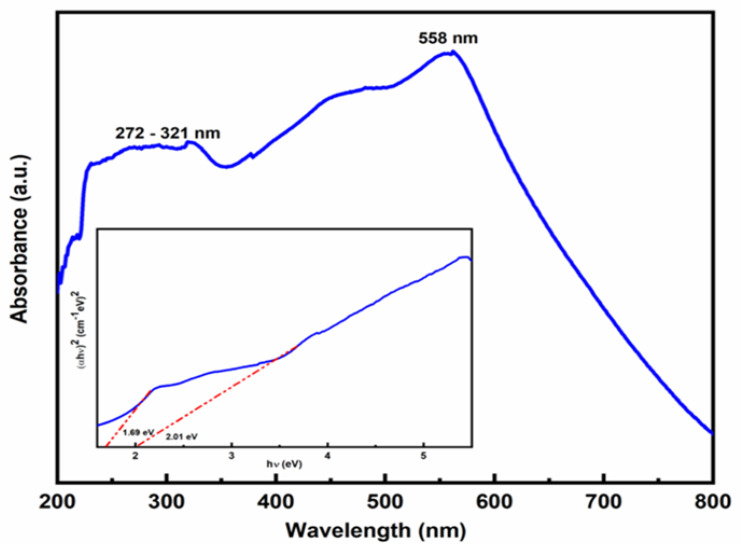
UV-vis absorption spectrum of Fe_2_O_3_ nanoparticles showing a conformational peak at 558 nm. The inset shows the plot of (αhv)^2^ vs. photon energy (hv) 1.69 eV and 2.01 eV due to the direct and indirect transition.

The synthesized sample’s UV-vis absorption spectrum was measured between 200 and 800 nm, and the related recorded data are presented in Figure 2. The peak at 558 nm in the visible region was attributed to the 6A1+6A1-4T1 (4G)+4T1 (4G) double excitation process of Fe^3+^, while the absorption band between 272 and 321 nm was due to the ligand-metal charge transfer transition (direct transition) and assigned to the 6A1-4T1 (4P) and 6A1-4T2 [29]. The twofold excitation process that gives hematite its red color causes the greatest absorption band to be visible at 558 nm [30].

The bandgap energy (Eg) of Fe_2_O_3_ nanoparticles was calculated using Tauc’s plot method by the formula

$$\alpha =c{\left(hv-E_{bulk}\right)}^{1/2}hv,$$

where *α* = absorption coefficient, *c* = constant, *hν* = photon energy, and *E_bulk_* = bulk bandgap. Scientists reported that Fe_2_O_3_ has an indirect bandgap as well as a direct bandgap [31]. The reported values of the indirect and direct bandgap lie between 1.38–2.09 eV [32] and 1.95–2.35 eV [33,34]. Here, the Fe_2_O_3_ nanoparticles’ bandgap energies were measured to be 1.69 and 2.01 eV, respectively. The energy of a photon is referred to as direct bandgap energy if the momentum of liberated holes in the valence band and electrons in the conduction band is the same. A photon cannot be released during the transition if an electron passes through an intermediate state, which is referred to as indirect bandgap energy. The quantum size effects of the nano-crystallites are responsible for the direct bandgap’s presence [35].

**Figure 3:**
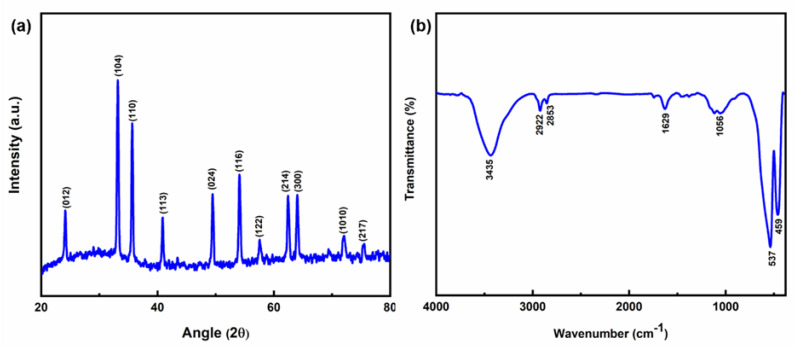
(**a**) The intense and sharp peaks in the XRD pattern of Fe_2_O_3_ nanoparticles show the phase purity of synthesized Fe_2_O_3_ nanoparticles. (**b**) FTIR spectra of Fe_2_O_3_ nanoparticles within the wavelength of 4000–400 cm^−1^.

The XRD spectrum of the prepared sample shown in Figure 3a denotes the crystallinity and the phase purity. The typical diffraction peaks were observed and matched by the JCPDS data (file no. 00-001-1053), confirming the synthesis of Fe_2_O_3_ nanoparticles. The sharp peaks at 24.14°, 33.16°, 35.66°, 40.9°, 49.48°, 54.08°, 57.56°, 62.46°, 64.04°, 72.04°, and 75.46° were associated with the plane (012), (104), (110), (113), (024), (116), (122), (214), (300), (1010), and (217), respectively, with a rhombohedral structure. The average crystalline size (D) of Fe_2_O_3_ was calculated by Debye–Scherer’s equation [36], $D=\frac{k\lambda }{\beta \cos\theta }$ where k = shape factor (0.89), *λ* = wavelength of Cu-Kα radiation (0.15406 nm), *β* = full width at half maximum (FWHM), *θ* = Bragg’s diffraction angle. From Scherer’s equation, the average size of the Fe_2_O_3_ nanoparticles is ~24.66 nm.

Figure 3b shows how FTIR was used to investigate the infrared characteristics of synthetic material in the wavelength range (4000–400 cm^−1^). Peaks associated with metal oxide bonds can be found in the fingerprint range between 1000 and 400 cm^−1^, whereas water molecule bending and stretching vibrations were responsible for peaks in the region between 400 and 1000 cm^−1^. The stretching vibration of the water molecules was what caused the absorption peak at 3435 cm^−1^ to be assigned [37]. The -OH stretching vibration was responsible for the peaks at 2922 and 2853 cm^−1^. Due to the bending vibration of the crystalline Fe-O bond and the absorbed moisture content, respectively, the peaks at 1629 and 1056 cm^−1^ were assigned [38]. The 537 and 459 cm^−1^ peaks were due to the vibration of Fe-O-Fe confirming the presence of Fe_2_O_3_ [39].

### 3.2. Morphology and Elemental Mapping of Fe_2_O_3_ Nanoparticles

**Figure 4:**
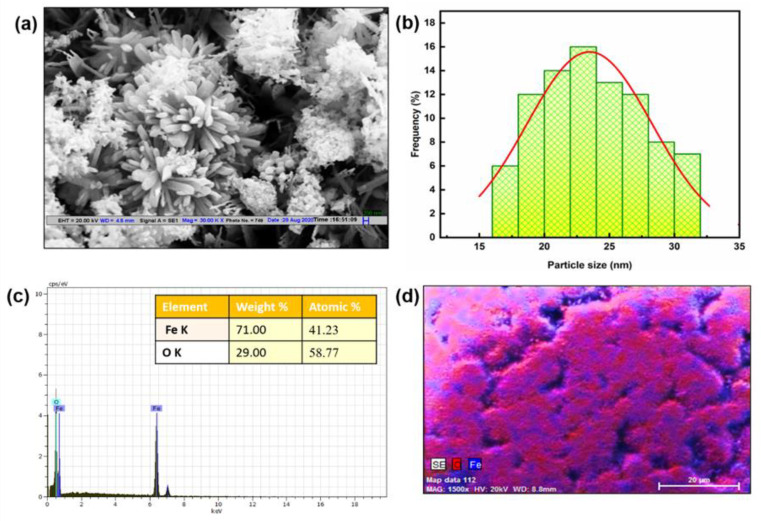
(**a**) FESEM image of Fe_2_O_3_ nanoparticles at 200 nm showing the flower-like morphology. (**b**) The histogram plot for the particle size distribution shows the size of Fe_2_O_3_ nanoparticles ~23.4 nm. (**c**) EDX spectrum shows the peaks of Fe and O atoms in nanoparticles. (**d**) Elemental mapping at 20 µm illustrated the distribution of present atoms in Fe_2_O_3_ nanoparticles.

Figure 4 depicts the shape of Fe_2_O_3_ nanoparticles that were studied using FESEM. The flower-like structure of Fe_2_O_3_ nanoparticles was visible in FESEM pictures (Figure 4a). According to FESEM pictures (Figure 4b), the nanostructures were about 23.4 nm in size, which was in agreement with the X-ray diffraction finding. Additionally, the components present in the Fe_2_O_3_ nanoparticles were confirmed by an EDX analysis, as shown in Figure 4c. The presence of the distinct peaks indicates the presence of Fe and O in the nanoparticles. Additionally, the purity of Fe_2_O_3_ nanoparticles was demonstrated by the peak of Fe and O devoid of any unknown signals. The distribution of components within Fe_2_O_3_ nanoparticles is depicted in Figure 4d.

**Figure 5:**
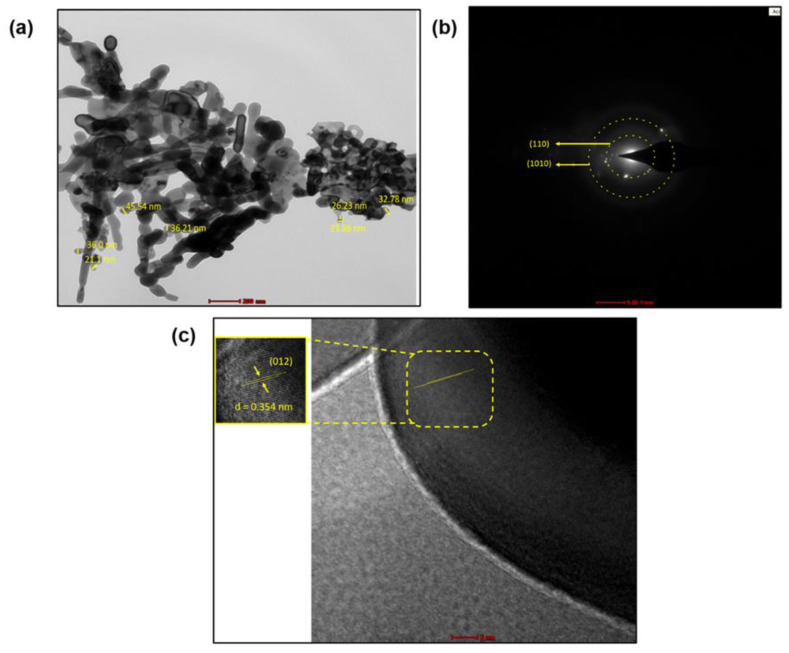
(**a**) HRTEM micrograph of Fe_2_O_3_ nanoparticles at 200 nm shows the size of nanoparticles between 22–45.5 nm (**b**) SAED pattern at 5 nm SAED pattern of Fe_2_O_3_ nanoparticles indicates crystallinity with different orientation (**c**) HRTEM image of Fe_2_O_3_ nanoparticles at 5 nm indicates high crystallinity and shows lattice fringes.

TEM images of Fe_2_O_3_ nanoparticles are shown in Figure 5. Due to the agglomeration, the size of the nanoparticles was seen as bigger. With the help of the Image J software, the calculated size of the Fe_2_O_3_ nanoparticles came out to be 22–45.5 nm (Figure 5a). The bright spot in the SAED pattern demonstrated the crystalline nature of Fe_2_O_3_ nanoparticles with different orientations (110), and (1010), respectively (Figure 5b). The growth direction of the Fe_2_O_3_ was parallel to the lattice fringes and the d-spacing of the (012) plane was about 0.354 nm, which was close to the lattice spacing of the rhombohedral Fe_2_O_3_ (Figure 5c). Considering our XRD study and close observation of the SAED images, it can be concluded that the diffraction rings indicate the rhombohedral Fe_2_O_3_ structure.

### 3.3. In Vitro Cytotoxicity Assay of Fe_2_O_3_ Nanoparticles

**Figure 6:**
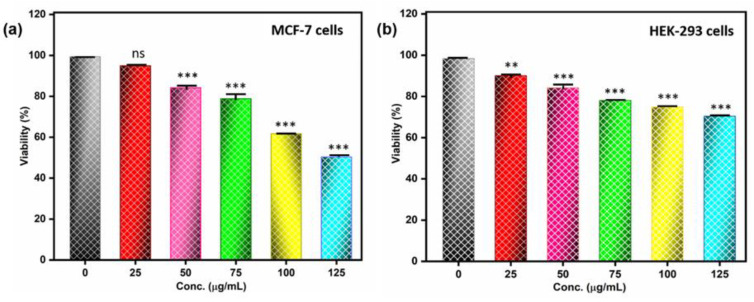
(**a**) Cell viability assay of Fe_2_O_3_ nanoparticles at different concentrations illustrated the dose-dependent cytotoxicity against the cancer cell line (MCF-7). (**b**) Cell viability assay against normal cell line (HEK-293) shows less toxicity as compared to cancer cell line. The graph representation showing the significant activity (*p* < 0.05 was considered to be statistically significant. The symbols **, *** represents, *p* < 0.01, *p* < 0.001).

The MTT test was used to investigate the metabolic effects of Fe_2_O_3_ nanoparticles on the MCF-7 and HEK-293 cell lines. The relevant answers demonstrated a percentage of cell viability after exposure to Fe_2_O_3_ nanoparticles at concentrations between 25 and 125 µg/mL for 24 h (Figure 6). In the MCF-7 cell line, cell viability was reduced to 50% when Fe_2_O_3_ nanoparticles were present at a concentration of 125 µg/mL (Figure 6a). The cytotoxicity of Fe_2_O_3_ nanoparticles against cancer cell lines supported the notion that nanoparticle cytotoxicity is dose-dependent. The normal embryonic kidney cell line (HEK-293) was also subjected to the MTT assay for 24 h, with results revealing less toxicity when compared to the breast cancer cell line (Figure 6b). The potential of Fe_2_O_3_ nanoparticles was demonstrated by the falling vitality of the cancer cell line in comparison to the normal cell line. Nanoparticles were reported to induce cytotoxic effects on human cells by employing multiple cell-mechanical approaches: (i) through the uptake of free nanoparticles causing defective DNA replication, (ii) through the generation of free radicals and reactive oxygen species (ROS), and (iii) by stressing the cell membrane, the structure of the entire cell membrane is deformed, followed by cell damage or cell death [40,41]. After ROS generation, altered mitochondrial membranes trigger caspase 3, a protein involved in organelle breakdown and DNA fragmentation, which, in turn, triggers apoptosis and cell cycle arrest. Apoptosis initiation leads to the activation of cell-signaling pathways, including activation of p53 protein, and increased p53 protein leads to cell death and nuclear destruction [42]. Moreover, the apoptosis pathways and method for cell death still need to be explained, through in vitro and in vivo studies.

Previously, researchers illustrated the cytotoxicity of Fe_2_O_3_ nanoparticles and nanocomposite of α-Fe_2_O_3_/Co_3_O_4_ on MCF-7 cell line for 24 h, showing that the nanocomposites are more toxic than α-Fe_2_O_3_ [43]. In another study, the cytotoxic efficiency on the A549 cell line was performed for 24 h and showed 50% cell viability at 970 µg/mL concentration [44]. However, our results showed that at 125 µg/mL concentration, 50% MCF-7 cell line were viable, indicating good cytotoxic activity of Fe_2_O_3_ nanoparticles. Based on the result, we can say that Fe_2_O_3_ nanoparticles can be used as a cytotoxic agent in cancer cell line

### 3.4. In Vitro Antibacterial Assessment of Fe_2_O_3_ Nanoparticles

**Figure 7:**
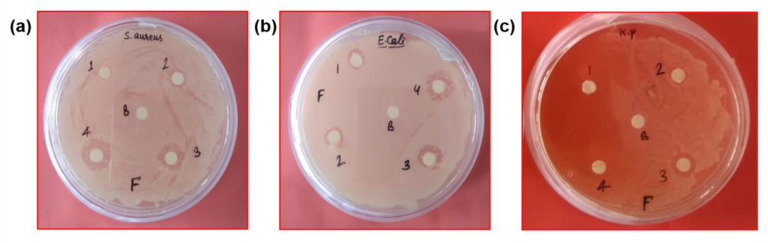
Antibacterial activity of Fe_2_O_3_ nanoparticles against Gram-positive and Gram-negative bacteria by disc diffusion method. The size of the zone of inhibition formed around each well of bacteria indicating the activity of Fe_2_O_3_ nanoparticles (**a**) *S. aureus* (**b**) *E. coli* (**c**) *K. pneumoniae* where (B-control, 1–10 µg/mL, 2–20 µg/mL, 3–30 µg/mL and 4–40 µg/mL).

The antibacterial action of synthesized Fe_2_O_3_ nanoparticles was conducted upon pathogenic bacteria, namely *S. aureus*, *E. coli*, and *K. pneumoniae*, employing the disc diffusion method. The inhibition zone showed the antibacterial activity of the Fe_2_O_3_ nanoparticles. The Fe_2_O_3_ nanoparticles revealed good inhibitory potential on bacterial pathogens (Figure 7). The probable mechanisms of bacterial cell death induced by the Fenton reaction were as reported in the literature [45]. After exposure to Fe_2_O_3_ nanoparticles to the pathogens, the iron ions released from nanoparticles can cross the membrane either through active uptake into cells or through leakage from sites of reduced membrane integrity. Highly reactive hydroxyl radicals formed when Fe^2+^ reacts with hydrogen peroxide primarily cause oxidative damage. Fe^3+^ can be reduced by NADH to regenerate Fe^2+^. OH radicals can also damage DNA, proteins, and lipids. Fe^2+^ can also directly damage DNA, resulting in cell death of pathogens [46].

**Figure 8:**
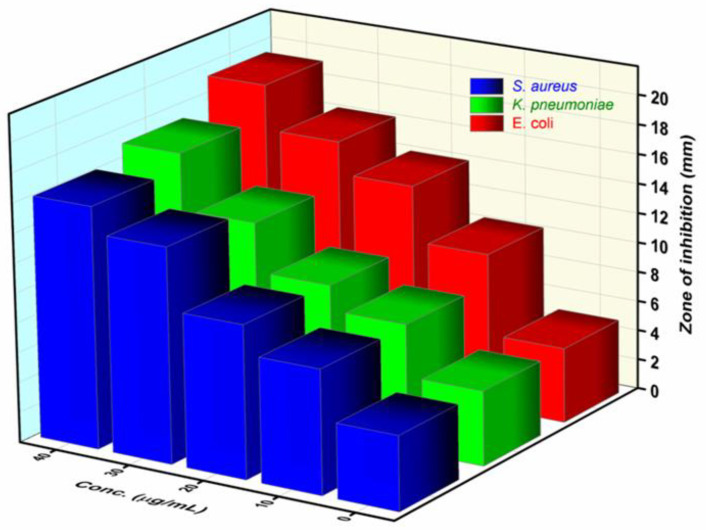
3D representation of the inhibition zone formed around respective discs of bacteria *S. aureus*, *K. pneumoniae*, and *E. coli* representing the activity of Fe_2_O_3_ nanoparticles.

Among three pathogenic bacteria, *E. coli* showed more activity than other bacteria. It was also shown that after increasing the dose of Fe_2_O_3_ nanoparticles, the inhibition zone increased, as shown in Figure 8 and Table 1. The observations reported that a smaller amount of Fe_2_O_3_ nanoparticles are sufficient for the activity against the tested bacterial strains. Our results are comparable with the results reported by Saquib and their co-workers [19].

**Figure 9:**
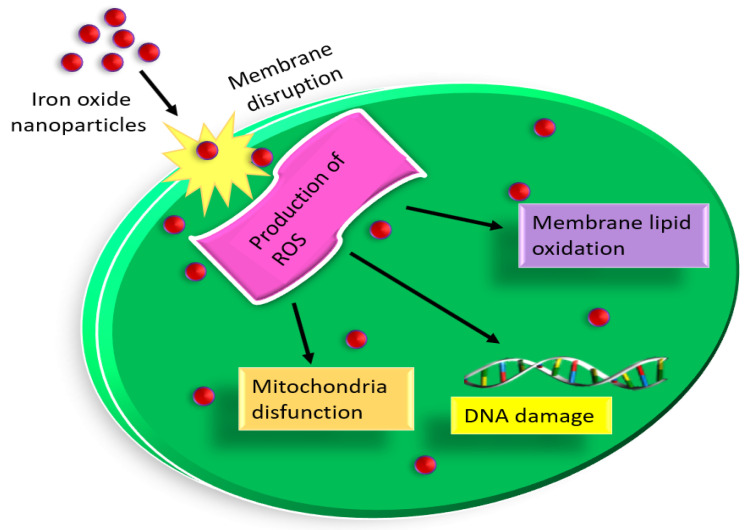
Schematic representation for a possible mechanism of Fe_2_O_3_ nanoparticles towards antibacterial activity. The effectiveness of Fe_2_O_3_ nanoparticles is based on the production of ROS, which is responsible for the cell death of microorganisms. ROS production cannot develop immunity because Since ROS attack many different sites and biomolecules in the microorganism, they cannot develop resistance resulting in oxidation and cell death.

Researchers discovered that ROS causes oxidative stress, which is why antibacterial medications exhibit bactericidal characteristics [47]. Additionally, several studies showed that ROS is crucial for cell signalling and death [48]. Due to the production of ROS, one of the most well-known silver nanoparticles exhibits antibacterial potential [49]. In our material, it was expected that Fe_2_O_3_ nanoparticles, which prevent microbial development, can produce ROS (Figure 9). The process by which hydrogen peroxide (H_2_O_2_) was produced when iron (Fe^2+^) and oxygen interacted was described by Keenan et al. [50]. Additionally, this H_2_O_2_ interacted with Fe^2+^ ions to create hydroxyl (OH^−^*) radicals, which damage macromolecules and membranes. Numerous studies showed that nanoparticles of various sizes can enter cells, interact with intracellular oxygen, and cause oxidative stress, which weakens the membrane [46,51,52]. Additionally, according to several studies, the concentration of nanoparticles was a crucial element in boosting antibacterial activity [53,54].

### 3.5. Antioxidant Efficiency of Fe_2_O_3_ Nanoparticles: A Possible Mechanism of Action for Antibacterial and Cytotoxic Activity

**Figure 10:**
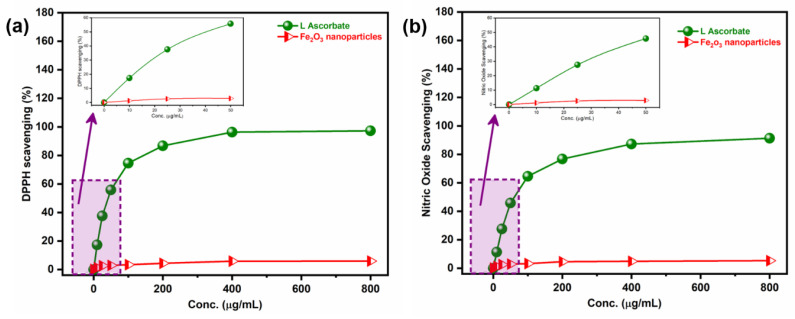
Antioxidant activity of Fe_2_O_3_ nanoparticles. (**a**) DPPH radical scavenging assay. (**b**) Nitric oxide scavenging assay showing the potential of synthesized Fe_2_O_3_ nanoparticles.

DPPH and nitric oxide (NO) scavenging assays were carried out to determine the antioxidant phenomenon of Fe_2_O_3_ nanoparticles (Figure 10). DPPH is a stable free radical delocalizing throughout the entire molecule to prevent its dimerization [55,56]. The DPPH was reduced to the formed stable, diamagnetic molecule when the nanoparticle was mixed with the solution resulting in changing the color of the solution from yellow to violet color. If the DPPH molecule is shown by X• and the donor molecule by ZH, the primary reaction is defined as X• + ZH = XH + Z*, where XH meant reduced form and Z* meant free radical. According to the UV-vis absorption curve in Figure 10a, the antioxidant potential of Fe_2_O_3_ nanoparticles was dose-dependent. The potential for inhibition in the production of nitrite with oxygen and oxides carried out the NO scavenging activity (Figure 10b). The overall findings demonstrated that at a concentration of 800 µg/mL, Fe_2_O_3_ nanoparticles exhibited high activity. The antibacterial and cell cytotoxic potential can be attributed to scavenging activity [57].

## 4. Conclusions

Since physiological processes occur at the nanoscale, many biological and medical issues are anticipated to be resolved through the use of nanotechnology and nanoparticles. To understand the mode of action and impact of various coatings to counteract the negative effect at the cellular level and optimize the potential of our nanoparticles for nanomedicine, a tailored investigation against each aspect is required. Our research revealed that Fe_2_O_3_ nanoparticle exposure to MCF-7 cells causes considerable cytotoxicity, opening up new possibilities for the safe delivery of Fe_2_O_3_ nanoparticles and their use in anticancer therapies. Additionally, it demonstrated another role in bacteria’s antimicrobial ability. Moreover, further research on the toxicity and biocompatibility aspects of animal models is also recommended to further understand their safety and biocompatible nature. Therefore, we may conclude that Fe_2_O_3_ nanoparticles can be employed as an acceptable substitute for an antibacterial drug that received clinical approval.

## Figures and Tables

**Table 1:** Antibacterial activity of synthesized Fe_2_O_3_ nanoparticles against the pathogen (* including disc size).

Concentration (µg/mL)	* Zone of Inhibition (mm) Mean ± SD		
	*S. aureus*	*K. pneumoniae*	*E. coli*
**Control**	5	5	5
**10**	8.3 ± 0.4	8.4 ± 0.4	10.4 ± 0.5
**20**	10.2 ± 0.2	10.1 ± 0.1	14.1 ± 0.1
**30**	14.4 ± 0.3	13.4 ± 0.4	16.3 ± 0.3
**40**	16.2 ± 0.2	17.2 ± 0.2	19.3 ± 0.3

## Data Availability

All data relevant to the publication are included.
